# Enhanced Localization in Wireless Sensor Networks Using a Bat-Optimized Malicious Anchor Node Prediction Algorithm

**DOI:** 10.3390/s24247893

**Published:** 2024-12-10

**Authors:** Balachandran Nair Premakumari Sreeja, Gopikrishnan Sundaram, Marco Rivera, Patrick Wheeler

**Affiliations:** 1 Department of Information Technology, Karpagam College of Engineering, Myleripalayam Village, Coimbatore 641032, Tamil Nadu, India; sreejabp@gmail.com; 2 School of Computer Science and Engineering, VIT-AP University, Amaravati 522237, Andhra Pradesh, India; gopikrishnan.s@vitap.ac.in; 3 Power Electronics, Machines and Control (PEMC) Research Institute, University of Nottingham, 15 Triumph Rd, Lenton, Nottingham NG7 2GT, UK; pat.wheeler@nottingham.ac.uk

**Keywords:** wireless sensor networks, localization, bat optimization, malicious nodes, clustering, probabilistic analysis

## Abstract

The accuracy of node localization plays a crucial role in the performance and reliability of wireless sensor networks (WSNs), which are widely utilized in fields like security systems and environmental monitoring. The integrity of these networks is often threatened by the presence of malicious nodes that can disrupt the localization process, leading to erroneous positioning and degraded network functionality. To address this challenge, we propose the security-aware localization using bat-optimized malicious anchor prediction (BO-MAP) algorithm. This approach utilizes a refined bat optimization algorithm to improve both the precision of localization and the security of WSNs. By integrating advanced optimization with density-based clustering and probabilistic analysis, BO-MAP effectively identifies and isolates malicious nodes. Our comprehensive simulation results reveal that BO-MAP significantly surpasses six current state-of-the-art methods—namely, the Secure Localization Algorithm, Enhanced DV-Hop, Particle Swarm Optimization-Based Localization, Range-Free Localization, the Robust Localization Algorithm, and the Sequential Probability Ratio Test—across various performance metrics, including the true positive rate, false positive rate, localization accuracy, energy efficiency, and computational efficiency. Notably, BO-MAP achieves an impressive true positive rate of 95% and a false positive rate of 5%, with an area under the receiver operating characteristic curve of 0.98. Additionally, BO-MAP exhibits consistent reliability across different levels of attack severity and network conditions, highlighting its suitability for deployment in practical WSN environments.

## 1. Introduction

Wireless sensor networks (WSNs) are integral to a wide range of applications, including environmental monitoring and security systems. These networks consist of numerous sensor nodes distributed across extensive areas, responsible for gathering and relaying data to central hubs for further analysis. The accuracy of the localization of the nodes, which is the precise determination of the geographical positions of these sensor nodes, is critical for the optimal operation of WSNs. Localization errors can pose significant challenges, such as inaccurate data interpretation, diminished network performance, and in extreme cases failure to meet mission objectives [1].

Given the frequent deployment of WSNs in challenging and often hostile environments, traditional localization techniques encounter considerable obstacles [2]. These conventional methods typically assume that the network environment is benign, with all nodes functioning correctly and without malicious interference. However, in real-world deployments, WSNs are vulnerable to attacks where malicious nodes are introduced into the network. These nodes can propagate false location information, thereby disrupting the localization process and threatening the overall security and reliability of the network [3].

The growing complexity of WSN applications, especially in security-critical areas, underscores the necessity for advanced localization algorithms that can perform reliably even in the presence of malicious nodes [4]. Although existing localization techniques have made strides in improving accuracy and efficiency, they often fall short when subjected to adversarial conditions. This highlights an urgent need for robust and secure localization methods that can withstand and counteract such threats [5].

### 1.1. Motivation and Challenges

The presence of malicious nodes within wireless sensor networks (WSNs) represents a significant threat to the accuracy and reliability of localization processes. These malicious entities have the ability to manipulate or falsify location information, resulting in incorrect node positioning and potentially compromising the entire network’s functionality. This issue is especially critical in applications like security systems, where precise localization is vital for the success of missions.

Recent advances in research have concentrated on improving the security and accuracy of WSN localization by incorporating optimization algorithms, such as Particle Swarm Optimization (PSO) and Genetic Algorithms (GAs) [6]. Although these techniques have demonstrated potential in enhancing localization accuracy, they still encounter obstacles related to computational efficiency and robustness, particularly when facing sophisticated and coordinated attacks [7].

To overcome these challenges, the proposed security-aware localization using bat-optimized malicious anchor prediction (BO-MAP) algorithm integrates a density-based clustering model with a bat-inspired optimization strategy. The BO-MAP algorithm is specifically designed to detect and isolate malicious nodes, thereby improving both the accuracy of localization and the security of the network. By combining the advantages of density-based clustering with probabilistic analysis, BO-MAP provides a more resilient and efficient solution for WSN localization in environments prone to adversarial threats [8].

### 1.2. Objective

The primary objective of this research is to address the significant challenges related to secure localization in WSNs by developing a robust and effective algorithm. This work aims to introduce a novel localization algorithm, named BO-MAP, which combines bat-inspired optimization with density-based clustering and probabilistic analysis. The integration of these techniques was designed to enhance the identification and exclusion of malicious nodes in WSNs, thereby improving both the accuracy of localization and the overall security of the network.

The comprehensive performance evaluation compares BO-MAP with several state-of-the-art localization methods, including the Secure Localization Algorithm (SLA) [9], Enhanced DV-Hop (EDV-Hop) [10], Particle Swarm Optimization-Based Localization (PSO-Loc) [11], Range-Free Localization (RFL) [12], the Robust Localization Algorithm (RLA) [13], and the Sequential Probability Ratio Test (SPRT). The evaluation focuses on key performance metrics such as the true positive rate (TPR), false positive rate (FPR), and overall localization accuracy.

Moreover, this research assesses the robustness of the BO-MAP algorithm under varying attack intensities and network conditions, demonstrating its potential applicability in real-world WSN deployments where both security and reliability are of utmost importance.

### 1.3. Contributions

This research provides several significant contributions to the field of WSN localization:

1. Introduction of the BO-MAP Algorithm and Its Robustness: We propose a novel algorithm, *security-aware localization using BO-MAP*, which integrates bat-inspired optimization with density-based clustering and probabilistic analysis. This innovative approach not only enhances localization accuracy and security by effectively detecting and excluding malicious nodes within WSNs but also maintains high localization performance and low false positive rates under varying attack intensities and diverse network conditions. This robustness highlights BO-MAP’s adaptability and reliability in real-world operational scenarios, significantly advancing the state of the art in secure WSN localization.
2. Thorough Performance Evaluation: Extensive simulations were conducted to assess the performance of the BO-MAP algorithm. These evaluations compared BO-MAP with six existing localization methods: the SLA, EDV-Hop, PSO-Loc, RFL, the RLA, and the SPRT. The results clearly demonstrate BO-MAP’s superior performance across multiple metrics, including the true positive rate (TPR), false positive rate (FPR), localization accuracy, energy efficiency, and execution time.
3. Implementation and Validation Guidance: We offer detailed guidelines for implementing the BO-MAP algorithm and validate its effectiveness through comprehensive simulation results. These practical insights facilitate the application of the algorithm in real-world WSNs, ensuring enhanced security and precision in the localization of nodes.

By addressing critical challenges in WSN localization [14] and offering a robust and efficient solution, this research significantly advances WSN technology and its application in various critical domains.

The remainder of this work is organized as follows. Section 2 reviews related work, including recent advancements in secure localization and optimization techniques for WSNs. Section 3 details the proposed BO-MAP methodology, covering the conceptual framework, network and communication model, attack detection model, clustering and optimization strategy, and algorithm implementation. Section 4 outlines the experimental setup and describes the evaluation metrics used in this study. Section 5 presents the simulation results, including comparisons with existing methods, robustness assessments, and sensitivity analyses. Finally, Section 6 concludes the paper with a summary of the contributions and suggestions for future research directions.

## 2. Related Work

Localization in wireless sensor networks (WSNs) has been a major research focus due to its essential role in enabling applications such as environmental monitoring and disaster management. Traditional localization techniques are generally categorized into range-based and range-free methods. Range-based methods, including the time of arrival (TOA), Angle of Arrival (AOA), and Received Signal Strength Indicator (RSSI), rely on measurements of the distance or angle between nodes to estimate positions. While these methods typically offer higher accuracy, they require additional hardware and are more energy-intensive [15].

However, range-free methods, such as Centroid Localization and DV-Hop, estimate node positions based on connectivity and relative distances between nodes without the need for precise measurements. Although these methods are less accurate, they are better suited for resource-constrained WSNs due to their lower computational and energy demands. Recent improvements in these methods have focused on enhancing accuracy and reducing energy consumption through various optimization techniques, such as Genetic Algorithms (GAs) and Particle Swarm Optimization (PSO).

### 2.1. Secure Localization Algorithms

With the increasing deployment of WSNs in critical and hostile environments, the demand for secure localization methods has become increasingly important. Traditional localization techniques often assume that all nodes within the network are trustworthy, which is not always the case in real-world scenarios. Malicious nodes can disrupt the localization process by providing false location information, leading to significant errors in the node positioning [16].

To combat this issue, various secure localization algorithms have been developed. For example, the Secure Localization Algorithm (SLA) incorporates cryptographic techniques to ensure the authenticity and integrity of localization data. The Enhanced DV-Hop (EDV-Hop) modifies the traditional DV-Hop algorithm by integrating security measures to detect and exclude malicious nodes. Similarly, Robust Localization Algorithms (RLAs) use redundancy and statistical methods to mitigate the impact of malicious nodes.

Probabilistic detection methods have also been employed to enhance the security of localization in WSNs. These methods utilize statistical models to estimate the likelihood of nodes being malicious based on their behavior or reported information. Techniques such as Bayesian inference and Markov Random Fields allow for the probabilistic modeling of node trustworthiness, enabling the network to isolate or mitigate the effects of malicious nodes [17,18].

Another approach is the Sequential Probability Ratio Test (SPRT), which employs statistical hypothesis testing to identify and isolate malicious nodes. Although these methods have shown potential in improving localization security, they often involve trade-offs in computational complexity and energy consumption, making them less suitable for resource-constrained WSNs.

### 2.2. Optimization Algorithms in WSN Localization

Optimization algorithms have been extensively used to improve the accuracy and efficiency of localization in wireless sensor networks (WSNs). Techniques such as Particle Swarm Optimization (PSO), Genetic Algorithms (GAs), and the Firefly Algorithm (FA) have been employed to optimize node placement, reduce localization errors, and minimize energy consumption [2].

PSO, inspired by the social behavior of flocking birds or schooling fish, is particularly popular due to its simplicity and effectiveness in finding near-optimal solutions. However, PSO can suffer from premature convergence, especially in complex search spaces, which can limit its effectiveness in highly dynamic WSN environments [19,20].

The GA, which leverages principles of natural selection and genetics, evolves solutions over generations and offers robustness against local optima. The GA has been successfully applied to various WSN localization problems, particularly those involving large search spaces. However, the GA can be computationally intensive, limiting its applicability in energy-constrained WSNs [21].

The FA, inspired by the flashing behavior of fireflies, has also been applied to WSN localization [22]. The FA is effective in addressing multimodal optimization problems and can adapt to dynamic network conditions. However, like other optimization algorithms, the FA requires careful parameter tuning to achieve optimal performance [23,24].

The Bat Algorithm (BA), inspired by the echolocation behavior of bats, has recently been applied to WSN localization. The BA simulates bats’ echolocation capabilities to search for optimal solutions, balancing exploration and exploitation in the search space. In WSN localization, the BA has shown advantages in the convergence speed and solution accuracy over traditional algorithms like PSO and GAs. Its ability to adjust frequency and loudness parameters allows the BA to navigate complex optimization landscapes effectively, making it suitable for dynamic WSN environments [25,26].

Building upon these existing optimization techniques, the proposed BO-MAP algorithm integrates the strengths of the Bat Algorithm with advanced clustering and probabilistic detection methods. BO-MAP addresses the limitations of traditional algorithms, such as premature convergence in PSO and high computational demands in GA, by introducing dynamic frequency modulation and adaptive parameter control. This integration enhances both the convergence speed and localization accuracy, ensuring robust performance in dynamic and hostile WSN environments. Additionally, BO-MAP incorporates density-based clustering and the Sequential Probability Ratio Test (SPRT) to effectively detect and exclude malicious nodes, thereby improving the overall security and reliability of the localization process.

### 2.3. Clustering Methods for WSNs

Clustering is another technique employed to enhance WSN localization. Clustering involves grouping nodes into clusters, each managed by a cluster head, which simplifies the localization process [27]. The Density-Based Spatial Clustering of Applications with Noise (DBSCAN) is a widely used clustering algorithm that identifies clusters based on the node density, making it suitable for WSNs where node distribution may be uneven.

The DBSCAN does not require a predefined number of clusters and can effectively identify and exclude outliers, making it robust against the presence of malicious nodes. However, the effectiveness of the DBSCAN is dependent on the appropriate selection of parameters, such as the minimum number of points required to form a cluster and the distance threshold [28].

Recent enhancements to the DBSCAN have improved its applicability in WSNs. Adaptive versions of the DBSCAN adjust parameters in response to changes in the node density and distribution, improving clustering accuracy in dynamic environments. Additionally, integrating the DBSCAN with optimization algorithms like the BA has led to improved cluster formation and energy efficiency, as the optimization algorithm can fine-tune cluster parameters for optimal performance [29,30].

Recent studies have combined clustering with optimization algorithms to further improve localization accuracy and security [31]. For example, clustering algorithms like the DBSCAN have been integrated with PSO and GAs to optimize cluster formation and improve the resilience of WSNs against attacks.

### 2.4. Research Gaps and Challenges

Despite considerable advancements, several challenges persist in achieving robust and efficient localization in wireless sensor networks (WSNs). The primary issues include the following.

- Computational Complexity: Secure localization methods often exhibit high computational complexity, particularly those employing cryptographic techniques or hybrid algorithms. This complexity poses a significant challenge for WSNs, where sensor nodes are constrained by limited processing power and energy resources. Algorithms that require extensive computations can rapidly deplete the battery life, reducing the overall operational time of the network.
- Sensitivity to Environmental Factors: The localization accuracy is highly vulnerable to environmental factors such as noise, signal attenuation, and multipath effects. Range-based methods are especially prone to these issues, leading to significant localization errors in dynamic environments. Methods like the Enhanced DV-Hop and Range-Free Localization also struggle with challenges related to node mobility and environmental changes.
- Scalability and Adaptability: Scalability remains a critical concern for WSNs deployed in large areas. Many existing methods find it difficult to maintain accuracy and efficiency as the size of the network increases. Additionally, adaptability to changing network conditions and the presence of malicious nodes is crucial for practical deployment but remains a significant challenge.
- Integration and Practical Implementation: Innovative approaches that integrate multiple optimization techniques are needed, harnessing their combined strengths to address individual limitations. Hybrid methods capable of dynamically adjusting to changing network conditions and malicious behaviors hold promise but require practical validation. Bridging the gap between theoretical models and real-world applications necessitates extensive field testing and practical implementations to ensure the applicability and effectiveness of these algorithms.

This review of existing localization methods in WSNs underscores both their strengths and limitations [32]. Although there have been significant advances in secure localization and optimization techniques, substantial challenges remain in developing methods that are robust, scalable, and secure against sophisticated attacks. The proposed BO-MAP algorithm seeks to address these gaps by integrating bat optimization with density-based clustering and probabilistic analysis, providing a comprehensive solution to accurate and secure localization in dynamic and potentially hostile environments [33].

This proposed approach addresses the aforementioned research gaps through the development of the BO-MAP algorithm, which integrates adaptive frequency-modulated bat optimization (AFM-BO), the Density-Based Spatial Clustering of Applications with Noise (DBSCAN), and the Sequential Probability Ratio Test (SPRT) for secure and accurate localization in WSNs. Specifically, BO-MAP reduces computational complexity by leveraging the efficiency of AFM-BO and the DBSCAN, enhances robustness to environmental factors through adaptive parameter control and dynamic frequency modulation, improves scalability and adaptability with its modular design and SPRT integration, and facilitates integration and practical implementation by combining optimization, clustering, and probabilistic detection techniques. These combined strengths address individual limitations and ensure that BO-MAP can be effectively implemented in real-world WSN deployments, as validated through extensive simulations and performance evaluations presented in subsequent sections.

## 3. Proposed Methodology

The BO-MAP system leverages the synergistic integration of AFM-BO, clustering, and probabilistic detection to create a secure and accurate localization framework. Unlike traditional optimization-based methods that treat all nodes uniformly, BO-MAP specifically identifies and excludes malicious nodes, thereby enhancing both the localization precision and network security. This targeted approach ensures that the optimization process is not compromised by adversarial nodes, leading to more reliable localization outcomes.

In comparison to existing optimization problem-solving techniques, which may not differentiate between legitimate and malicious nodes, BO-MAP employs a multi-faceted strategy that combines optimization with clustering and statistical testing. This comprehensive methodology not only optimizes the localization accuracy but also fortifies the network against security threats, making it a significant advancement in the field of WSNs.

### 3.1. Problem Formulation

Wireless sensor networks (WSNs) are extensively utilized in various applications, including security systems and environmental monitoring. The accuracy of node localization is paramount to the performance and reliability of these networks. However, the presence of malicious nodes poses a significant threat, potentially disrupting the localization process and degrading the overall network functionality. This research aims to enhance both the accuracy and security of node localization in WSNs by effectively identifying and mitigating the impact of malicious nodes.

#### 3.1.1. Problem Statement

The primary objective of this research is to develop an optimization-based localization algorithm that minimizes the localization error while ensuring the security of WSNs against malicious anchors. The problem is formulated as an optimization task where the goal is to minimize the Root Mean Square Error (RMSE) between the estimated and actual positions of sensor nodes, simultaneously detecting and excluding malicious nodes from the localization process.

Previous studies have addressed localization accuracy in WSNs through various optimization techniques. For instance, the DV-Hop algorithm [34] and its enhanced versions [35] have been widely used for range-based localization. However, these methods often assume a benign network environment and do not account for the presence of malicious nodes, which can significantly impair localization accuracy [36]. To address these limitations, security-aware localization algorithms have been proposed, integrating mechanisms to detect and mitigate the impact of malicious anchors [37,38].

#### 3.1.2. Objective Function

Given a set of anchor nodes $A=\{A_{1},A_{2},\ldots ,A_{n}\}$ with known positions and a set of sensor nodes $S=\{S_{1},S_{2},\ldots ,S_{m}\}$ with unknown positions, the objective is to accurately determine the positions $\hat{S}=\left\{{\hat{S}}_{1},{\hat{S}}_{2},\ldots ,{\hat{S}}_{m}\right\}$ of the sensor nodes while minimizing the impact of malicious anchors.

The localization error *E* is quantified using the Root Mean Square Error (RMSE) between the estimated and actual positions of the sensor nodes:

(1) $$E=\sqrt{\frac{1}{m}\sum_{i=1}^{m}{∥{\hat{S}}_{i}-S_{i}∥}^{2}}$$

The objective is to minimize this error *E*, subject to various constraints imposed by network conditions and the presence of malicious nodes. The BO-MAP algorithm, enhanced by adaptive frequency-modulated bat optimization (AFM-BO), aims to find the optimal position estimates $\hat{S}$ while identifying and mitigating the impact of malicious nodes, denoted by $M=\{M_{1},M_{2},\ldots ,M_{k}\}$.

Previous optimization-based approaches, such as Particle Swarm Optimization (PSO) [39] and Genetic Algorithms (GAs) [40], have been employed to enhance localization accuracy. However, these methods often require extensive computational resources and may not effectively handle dynamic network conditions or the presence of multiple malicious nodes [41]. The proposed AFM-BO seeks to overcome these challenges by introducing dynamic frequency modulation and adaptive parameter control, thereby improving both exploration and exploitation capabilities during the optimization process.

#### 3.1.3. Constraints

The optimization problem is subject to several constraints that ensure the feasibility and robustness of the localization process:

- Communication Range: Sensor nodes can only communicate with anchor nodes within a certain distance $d_{\max}$ from them. This constraint limits the set of anchor nodes that can be utilized for localization [39]. Effective localization requires sufficient anchor coverage to ensure that each sensor node can communicate with multiple anchors, thereby enhancing localization accuracy [35].
- Malicious Nodes: The presence of malicious nodes *M*, which provide false position information, must be detected and their influence excluded from the localization process to maintain the network’s integrity [37,38]. Malicious nodes can manipulate distance measurements or provide fabricated data, leading to significant localization errors if not properly identified and mitigated [38].
- Environmental Noise: Measurement errors due to environmental noise, modeled as Gaussian noise with variance $\sigma^{2}$, affect the accuracy of distance estimates between nodes [40]. Environmental factors such as signal attenuation, multipath effects, and interference can introduce variability in the Received Signal Strength Indicator (RSSI) and time of arrival (TOA) measurements, thereby impacting the localization precision [41].

The BO-MAP algorithm, utilizing AFM-BO, was designed to optimize the localization process under these constraints by leveraging dynamic frequency modulation, adaptive parameter control, clustering, and probabilistic detection to mitigate the impact of malicious nodes.

#### 3.1.4. Optimization Approach

To achieve the objectives and navigate the constraints outlined above, the BO-MAP algorithm integrates three key components:

- Adaptive Frequency-Modulated Bat Optimization (AFM-BO): AFM-BO enhances the traditional bat optimization algorithm by introducing dynamic frequency modulation and adaptive parameter control. This optimization technique effectively balances exploration and exploitation phases, enabling the algorithm to dynamically adjust the search process based on the current solution’s fitness [42]. Consequently, AFM-BO improves both the precision of localization and the efficiency of the optimization process. Unlike standard optimization algorithms, AFM-BO can adapt to the evolving landscape of the solution space, making it particularly effective in dynamic and hostile network environments [43].
- Clustering with the DBSCAN: Clustering was employed to group sensor nodes and identify outliers (potentially malicious nodes) based on their distance metrics. The Density-Based Spatial Clustering of Applications with Noise (DBSCAN) algorithm was utilized for this purpose due to its effectiveness in identifying clusters of arbitrary shapes and handling noise [44]. A modified version of the DBSCAN was integrated to adaptively select clustering parameters based on the node density and variance, thereby enhancing the detection of malicious nodes in dynamic network conditions [45]. This adaptive clustering approach ensures that legitimate clusters are accurately identified while isolating anomalous nodes that may exhibit malicious behavior [46].
- Probabilistic Detection via the SPRT: The Sequential Probability Ratio Test (SPRT) was implemented to statistically evaluate and exclude malicious nodes from the localization process. The SPRT enables the real-time assessment of node legitimacy by continuously monitoring discrepancies in distance measurements and updating the likelihood of malicious behavior [47]. This probabilistic approach ensures the integrity of the localization process by effectively isolating malicious nodes. By integrating the SPRT with AFM-BO and DBSCAN clustering, BO-MAP provides a robust framework for secure and accurate node localization in the presence of adversarial threats [48].

The integration of AFM-BO with clustering and probabilistic detection techniques allows the BO-MAP algorithm to dynamically adapt to varying network conditions and effectively mitigate the impact of malicious nodes. This comprehensive approach ensures that localization accuracy is maximized while maintaining the **integrity** and **security** of the network. Compared to traditional optimization methods, which may treat all nodes uniformly without accounting for malicious behavior, BO-MAP selectively optimizes the localization process by identifying and excluding harmful nodes, thereby enhancing both performance and security [48].

### 3.2. Overview of the BO-MAP Model

The BO-MAP model was designed to enhance both the accuracy and security of node localization in WSNs. This model integrates the advanced adaptive frequency-modulated bat optimization algorithm (AFM-BO) with clustering and detection techniques to effectively identify and mitigate the influence of malicious anchor nodes. The primary objective of BO-MAP is to ensure precise localization while minimizing errors, making it particularly suitable for deployment in environments where the network may be subject to adversarial activities.

The BO-MAP model introduces several key innovations. The AFM-BO algorithm, an enhanced version of the traditional bat optimization algorithm, incorporates dynamic frequency modulation and adaptive parameter control. These enhancements optimize the balance between exploration and exploitation during the search process, leading to more accurate and efficient localization. Furthermore, the model employs dynamic clustering techniques to identify and isolate malicious nodes, thus ensuring the robustness of the localization process. The probabilistic detection of malicious nodes is conducted using the Sequential Probability Ratio Test (SPRT), which enables the real-time identification of malicious nodes and maintains the reliability of the network’s spatial information.

### 3.3. Network and Communication Model

**Figure 1:**
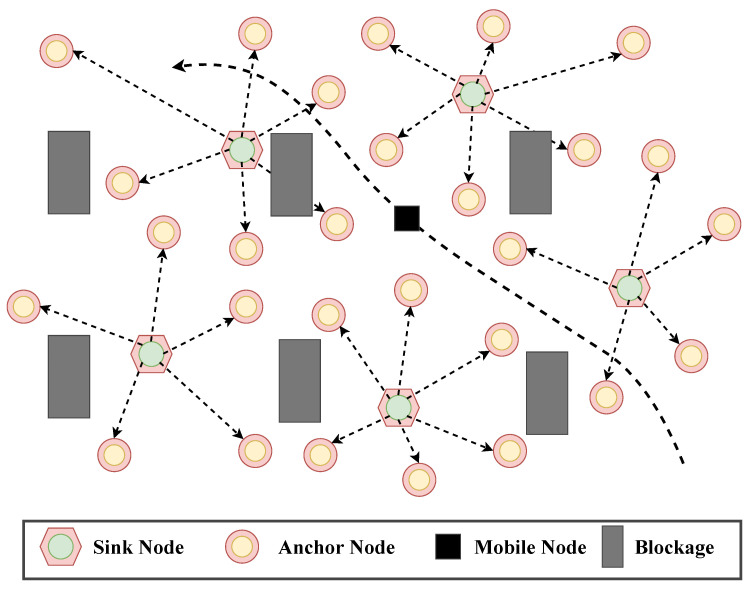
Node deployment in wireless sensor network.

In WSNs, nodes are deployed in a defined area where they communicate wirelessly to perform various tasks such as environmental monitoring, security surveillance, and disaster management. The network is modeled as a two-dimensional plane with sensor and anchor nodes distributed throughout the area. The positions of the anchor nodes are indicated by coordinates $A_{i}=\left[x_{i},y_{i}\right]$, while the unknown nodes are represented by $\Theta =[x_{\theta },y_{\theta }]$. The anchor nodes are assumed to be fixed, and the unknown nodes need to be localized based on the signals they receive from the anchors. As shown in Figure 1, the network includes various types of nodes such as sink nodes, anchor nodes, and mobile nodes; some areas are potentially blocked due to environmental factors or obstacles. These blockages can significantly affect the communication between nodes and therefore affect the accuracy of localization.

The communication model involves the exchange of signals that are used to estimate distances, ultimately determining the location of the unknown nodes. The BO-MAP model employs two primary techniques for distance estimation: the TOA and RSSI.

The TOA measures the time it takes for a signal to travel from an anchor node to an unknown node. The distance $d(\Theta ,A_{i})$ between an unknown node Θ and the *i*-th anchor node $A_{i}$ is calculated using the equation

(2) $$t_{i}=\frac{d(\Theta ,A_{i})}{v_{p}}+W_{i}$$

where the symbols correspond to the following:

- $t_{i}$: the measured time of arrival of the signal from the anchor node $A_{i}$ to the unknown node Θ.
- $d(\Theta ,A_{i})$: the Euclidean distance between the unknown node Θ and the *i*-th anchor node $A_{i}$, which is the parameter being estimated.
- $v_{p}$: the propagation speed of the signal, which is typically assumed to be the speed of light in free space (approximately $3\times 10^{8}$ m/s).
- $W_{i}$: the measurement error, modeled as a Gaussian random variable $W_{i}∼N\left(0,\sigma_{W}^{2}\right)$ with zero mean and variance $\sigma_{W}^{2}$, accounting for inaccuracies due to environmental factors (e.g., noise, obstacles, multipath effects) and equipment limitations.

The equation models the relationship between the time of arrival, the distance, and the propagation speed, incorporating the uncertainty introduced by real-world measurement errors.

The RSSI estimates the distance based on the power of the received signal. The relationship between the received power $P_{R}$ and the distance $d(\Theta ,A_{i})$ is given by

(3) $$P_{R}=P_{T_{i}}-10\alpha \log\left(\frac{d(\Theta ,A_{i})}{d_{0}}\right)+\epsilon_{i}$$

where the symbols correspond to the following:

- $P_{T_{i}}$ is the transmitted power from the anchor node $A_{i}$.
- α is the path loss exponent that characterizes the rate at which the signal attenuates with distance.
- $d_{0}$ is a reference distance (typically 1 m).
- $\epsilon_{i}∼N\left(0,\sigma_{\epsilon }^{2}\right)$ accounts for measurement noise, where $N(0,\sigma_{\epsilon }^{2})$ denotes a Gaussian distribution with a mean of zero and a variance of $\sigma_{\epsilon }^{2}$. The mean represents the expected value (no bias in measurement noise), and the variance quantifies the spread or variability of the noise.

The localization process begins by collecting distance measurements using TOA and RSSI techniques. An initial position estimate for the unknown node is derived from these measurements. However, due to potential measurement errors and the presence of malicious nodes, this initial estimate may be inaccurate. The BO-MAP model refines the localization using optimization and clustering techniques, which enhance accuracy by mitigating the impact of erroneous or malicious data.

### 3.4. Attack Model

In the BO-MAP model, two primary categories of attacks are considered: location data tampering and signal spoofing.

- Location Data Tampering: A malicious anchor node provides false location information, disrupting the localization process and causing significant errors. The model detects such tampering by analyzing data consistency using clustering techniques, identifying outliers that may indicate malicious activity.
- Signal Spoofing: A more sophisticated attack where the signal characteristics, such as timestamps or the transmission power, are altered by a malicious node to deceive the localization process. The TOA-based distance estimation was modified to account for this
  (4) $$t_{i}=\frac{d(\Theta ,A_{i})}{v_{p}}+W_{i}+\delta_{i}$$
  where the symbols correspond to the following:
  - $t_{i}$ is the measured time of arrival.
  - $v_{p}$ is the propagation speed of the signal (assumed to be the speed of light).
  - $W_{i}∼N\left(0,\sigma_{W}^{2}\right)$ represents measurement noise due to environmental factors, modeled as a Gaussian distribution with mean zero (unbiased) and variance $\sigma_{W}^{2}$ (quantifying noise variability).
  - $\delta_{i}∼N\left(\mu_{\delta },\sigma_{\delta }^{2}\right)$ represents the malicious delay introduced by the attacker, where $\mu_{\delta }$ is the expected malicious delay and $\sigma_{\delta }^{2}$ quantifies its variability.

These attack models provide a framework for understanding potential threats and guide the development of detection mechanisms to ensure robust localization.

#### Attack Model and Its Integration

The proposed BO-MAP algorithm accounts for the presence of malicious anchor nodes, which are modeled as nodes providing deliberately falsified localization data. These malicious nodes exploit vulnerabilities by either manipulating time-based (TOA) or signal-strength-based (RSSI) measurements, or by injecting spurious data into the network. To address these adversarial conditions, the BO-MAP algorithm integrates the attack model into its core components:

- Clustering-Based Outlier Detection: The DBSCAN clustering technique is used to identify high-density regions of normal data and isolate outliers, which often correspond to malicious nodes. Adaptive parameter tuning ensures the algorithm remains robust across varying attack intensities.
- Statistical Detection via the SPRT: The Sequential Probability Ratio Test (SPRT) evaluates deviations in distance measurements provided by anchor nodes. Statistical thresholds, informed by the attack model, are dynamically adjusted to detect anchors that consistently provide anomalous data.
- Optimization Adaptation: The adaptive frequency-modulated bat optimization (AFM-BO) algorithm leverages the attack model to prioritize optimization paths that exclude data from suspected malicious nodes, thereby minimizing localization errors.

The attack model was further utilized in simulation scenarios to evaluate the algorithm’s performance under varying proportions of malicious nodes, attack intensities, and environmental noise levels. This integration ensures that BO-MAP maintains high localization accuracy and security, even in hostile WSN environments.

### 3.5. Clustering and Optimization

#### 3.5.1. Clustering Approach

The BO-MAP model employs a modified version of the Density-Based Spatial Clustering of Applications with Noise (DBSCAN) algorithm to better suit the unique characteristics of wireless sensor networks (WSNs) and to enhance malicious node detection. In the traditional DBSCAN, two critical parameters are required: the minimum number of points to form a cluster (MinPts) and the neighborhood radius (ϵ). Clusters are formed by connecting points that are within ϵ distance from each other and have at least MinPts neighbors. While the standard DBSCAN effectively identifies clusters based on the point density and can handle noise, it does not account for the dynamic and irregular node distributions commonly found in WSNs, nor does it incorporate any trust or security considerations.

Our modified DBSCAN algorithm introduces an adaptive mechanism for selecting the ϵ and MinPts parameters based on the local node density and the variance in node positions ($V_{r}$). Specifically, we calculate the neighborhood radius ϵ using the variance of the nodes’ positions ($V_{r}$), which allows the algorithm to adjust to areas of varying node density:

(5) $$\epsilon =\kappa \times V_{r}$$

where κ is a scaling factor determined empirically. The variance $V_{r}$ is computed as

(6) $$V_{r}=\frac{1}{N}\sum_{i=1}^{N}\left({\left(x_{i}-\bar{x}\right)}^{2}+{\left(y_{i}-\bar{y}\right)}^{2}\right)$$

Here, $\left(x_{i},y_{i}\right)$ represents the position of node *i*, and $\left(\bar{x},\bar{y}\right)$ is the mean position of all nodes. By adapting ϵ based on $V_{r}$, the algorithm becomes more sensitive to the actual distribution of nodes, improving cluster formation accuracy in heterogeneous networks.

Additionally, we integrated a trust metric into the clustering process. Each node is assigned a trust level based on its communication behavior and data consistency. During clustering, nodes with trust levels below a certain threshold are treated as noise, effectively isolating potential malicious nodes from legitimate clusters. This integration enhances the security of the localization process by preventing malicious nodes from influencing the cluster formation and subsequent localization computations.

**Figure 2:**
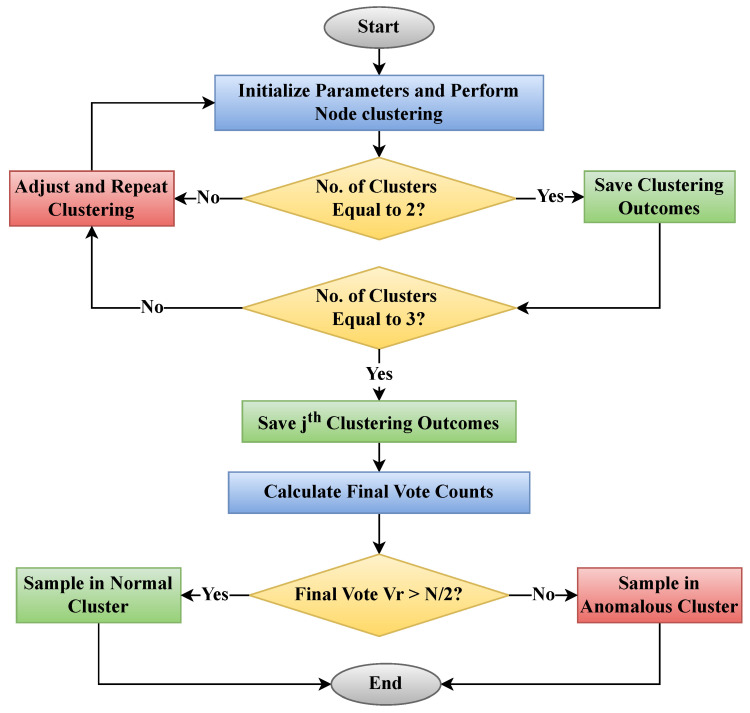
Process flow of clustering model.

In Figure 2, the no. of clusters equaling two signifies that the modified DBSCAN algorithm has identified two distinct clusters within the network based on the adapted parameters. This outcome demonstrates the algorithm’s ability to detect the inherent grouping of nodes in the WSN, which is essential for efficient localization and communication. The parameter $V_{r}$ represents the variance of the nodes’ positions and is crucial in dynamically adjusting the neighborhood radius ϵ for clustering. By incorporating $V_{r}$, the algorithm accounts for the spatial dispersion of nodes, leading to more accurate and context-aware clustering results.

The clustering process begins by identifying high-density regions within the network, which are then expanded to form clusters. These clusters represent groups of nodes that are close in proximity, and the method ensures that outlier nodes that do not belong to any cluster are effectively isolated. These outliers are often indicative of malicious nodes or errors in data, and their identification is crucial for maintaining the accuracy and security of the localization process. The adaptive nature of the clustering process allows it to dynamically adjust to the characteristics of the data, ensuring that the clustering results are robust against variations in the network density and topology.

#### 3.5.2. Optimization Strategy

The optimization process within the BO-MAP model is driven by the adaptive frequency-modulated bat optimization (AFM-BO) algorithm, which is tailored to enhance the accuracy of node localization in WSNs. The algorithm mimics the echolocation behavior of bats, where their movements are governed by the frequency, velocity, and position, dynamically updated based on the fitness of the current solution.

##### Frequency Adjustment

At each iteration, the frequency $f_{i}$ of each bat is adjusted to fine-tune the exploration of the search space. This adjustment is based on a random factor β, drawn from a uniform distribution within [0,1], ensuring diverse exploration:

(7) $$f_{i}=f_{\min}+\left(f_{\max}-f_{\min}\right)\times \beta$$

Here, the symbols correspond to the following:

- $f_{\min}$ and $f_{\max}$ are the minimum and maximum frequency bounds, controlling the bat’s step size.
- β is a random number in the range of [0,1] that introduces stochasticity to prevent premature convergence.

##### Velocity Update

The velocity $v_{i}\left(t+1\right)$ of each bat is updated based on the current position of the bat $x_{i}\left(t\right)$ and the best known position $x_{\mathrm{best}}$. This update helps the bats move toward promising regions in the search space:

(8) $$v_{i}\left(t+1\right)=v_{i}\left(t\right)+\left(x_{i}\left(t\right)-x_{\mathrm{best}}\right)\times f_{i}$$

Here, the symbols correspond to the following:

- $v_{i}\left(t\right)$ and $v_{i}\left(t+1\right)$ are the velocities of the *i*th bat at iterations of *t* and t+1, respectively.
- $x_{i}\left(t\right)$ is the current position of the *i*th bat, and $x_{\mathrm{best}}$ is the best position found so far.
- $f_{i}$ is the frequency determined in the previous step.

##### Position Update

Once the velocity is updated, the new position $x_{i}\left(t+1\right)$ of each bat is calculated by adding the updated velocity to the current position:

(9) $$x_{i}\left(t+1\right)=x_{i}\left(t\right)+v_{i}\left(t+1\right)$$

Here, the symbols correspond to the following:

- $x_{i}\left(t+1\right)$ is the updated position of the *i*th bat.
- $v_{i}\left(t+1\right)$ is the updated velocity of the bat.

##### Exploration and Exploitation

To balance exploration (searching new areas) and exploitation (refining known good areas), the algorithm adjusts the pulse rate $r_{i}\left(t+1\right)$ and loudness $A_{i}\left(t+1\right)$ of each bat. These adjustments encourage the bats to focus on a local search as they approach the optimal solution:

(10) $$r_{i}\left(t+1\right)=r_{i}\left(0\right)\times \left[1-\exp(-\gamma \times t)\right]$$

Here, the symbols correspond to the following:

- $r_{i}\left(0\right)$ is the initial pulse rate of the *i*th bat.
- γ is the exponential decay factor, controlling the rate at which the pulse rate increases.
- *t* is the current iteration number.

The loudness $A_{i}\left(t+1\right)$ diminishes over time to refine the search around the best solution:

(11) $$A_{i}\left(t+1\right)=\alpha \times A_{i}\left(t\right)$$

Here, the symbols correspond to the following:

- α is a constant reduction factor (0<α<1).
- $A_{i}\left(t\right)$ is the loudness of the *i*th bat at iteration *t*.

In addition to the global search, the AFM-BO algorithm incorporates a local search mechanism. When certain conditions are met, a bat performs a local search around the best known solution $x_{\mathrm{best}}$, introducing small perturbations to its position:

(12) $$x_{i}=x_{\mathrm{best}}+\epsilon \times A_{i}\left(t\right)$$

where ϵ is a random number drawn from a Gaussian distribution.

The process continues iteratively until a termination criterion is met, such as a predefined number of iterations or the convergence of solutions. Throughout the optimization process, the AFM-BO algorithm adapts dynamically to the evolving landscape of the solution space, making it particularly effective in environments where the presence of malicious nodes requires robust and adaptive optimization strategies.

Finally, the best solution identified by the bats is used in conjunction with the modified DBSCAN clustering algorithm to refine the localization of nodes and detect malicious nodes during the clustering process. The integration of AFM-BO with adaptive clustering and probabilistic detection techniques ensures that the BO-MAP model achieves high localization accuracy and security, even in challenging WSN environments.

### 3.6. Algorithm Implementation

#### Pseudocode of BO-MAP Algorithm

The pseudocode for the BO-MAP algorithm integrates the steps for both clustering and optimization, Aglorithm 1:
**Algorithm 1** AFM-BO Algorithm  1:Initialize bat population with random positions and velocities.  2:Define initial pulse rate $r_{i}$ and loudness $A_{i}$ for each bat.  3:**while** termination criterion not met **do**  4:    **for** each bat *i* in the population **do**  5:        Update frequency $f_{i}$ based on current solution fitness:
$$f_{i}=f_{\min}+\left(f_{\max}-f_{\min}\right)\times F\left(x_{i}\right)$$  6:        Update velocity $v_{i}\left(t+1\right)$ and position $x_{i}\left(t+1\right)$:
$$v_{i}\left(t+1\right)=v_{i}\left(t\right)+\left(x_{i}\left(t\right)-x_{\mathrm{best}}\right)\times f_{i}$$
$$x_{i}\left(t+1\right)=x_{i}\left(t\right)+v_{i}\left(t+1\right)$$  7:        **if** rand $<r_{i}$ **then**  8:           Perform local search around the best solution:
$$x_{i}=x_{\mathrm{best}}+\epsilon \times A_{i}\left(t\right)$$  9:        **end if**10:        **if** rand $<A_{i}$ and new solution is better **then**11:           Accept the new solution:
$x_{i}=x_{i}\left(t+1\right)$12:           Update pulse rate and loudness:
$$r_{i}\left(t+1\right)=r_{i}\left(0\right)\times \left[1-\exp\left(-\gamma \times t\right)\right]$$
$A_{i}\left(t+1\right)=\alpha \times A_{i}\left(t\right)$13:        **end if**14:    **end for**15:    Apply DBSCAN to cluster nodes based on bat positions.16:    Use SPRT to detect and exclude malicious nodes.17:**end while**18:Output the best solution.

The BO-MAP model offers a comprehensive solution for secure and accurate localization in WSNs. By integrating adaptive optimization, dynamic clustering, and probabilistic detection techniques, the model ensures robust performance even in the presence of malicious nodes. The detailed methodology outlined above highlights the innovative approaches employed to achieve high localization accuracy and enhanced network security in dynamic and potentially hostile environments.

### 3.7. Detection Model

In the BO-MAP system, the detection model plays a crucial role in identifying and mitigating the impact of malicious anchor nodes. This model relies on statistical analysis to detect anomalies in distance measurements, which could indicate malicious behavior.

The process begins by considering an arbitrary, unidentified node represented by Θ. Suppose there are *n* anchor nodes within the transmission range of Θ. The distance estimations between Θ and the $i^{th}$ anchor node are calculated using the RSSI and TOA methods. These estimations are denoted as $d_{rij}$ (RSSI-based) and $d_{tij}$ (TOA-based), respectively. The discrepancy between these two measurements for the $i^{th}$ anchor is represented by

(13) $$D_{ij}=d_{tij}-d_{rij}$$

where $D_{ij}$ signifies the difference between the TOA and RSSI measurements for the $j^{th}$ measurement.

To determine whether an anchor node is acting maliciously, the model calculates the variance in the reference error interval $D_{i}$, which is the difference between the RSSI measurement and the TOA measurement of the $i^{th}$ reference anchor:

(14) $$D_{i}=d_{ti}-d_{ri}$$

Given that the unidentified node Θ has *n* anchors within its transmission range, and for each anchor *i* there are $m_{i}$ measurement errors, the mean $\bar{D_{i}}$ and variance $s_{i}^{2}$ of the discrepancies between the two sets of metrics are calculated as follows:

(15) $$\bar{D_{i}}=\frac{\sum_{j}D_{ij}}{m_{i}}$$

(16) $$s_{i}^{2}=\frac{\sum_{j}{\left(D_{ij}-\bar{D_{i}}\right)}^{2}}{m_{i}-1}$$

The overall variance for the anchor measurements, considering all anchors within the transmission range, is derived as

(17) $$\bar{D}=\frac{\sum_{i}\bar{D_{i}}}{n}$$

The model employs the Bland–Altman technique to define the limits of agreement (LOAs), which are used to determine the confidence interval within which the measurements are considered acceptable. The LOA is calculated as follows:

(18) $$LOA_{l}=\bar{D}-z_{\left(1-\alpha /2\right)}\times \sqrt{s_{t}^{2}}$$

(19) $$LOA_{u}=\bar{D}+z_{\left(1-\alpha /2\right)}\times \sqrt{s_{t}^{2}}$$

where the symbols correspond to the following:

- $LOA_{l}$ and $LOA_{u}$ represent the lower and upper bounds of the confidence interval, respectively.
- $z_{\left(1-\alpha /2\right)}$ is the critical value of the standard normal distribution corresponding to the significance level α.
- $s_{t}^{2}$ is the generalized estimation of variance, given by

(20) $$s_{t}^{2}=s_{\epsilon }^{2}+\left(1-\frac{1}{m_{h}}\right)s_{a}^{2}$$

where $s_{\epsilon }^{2}$ represents the measurement noise variance, $s_{a}^{2}$ represents the anchor-related variance, and $m_{h}$ is the harmonic mean of the number of samples.

To enhance the detection process, the Sequential Probability Ratio Test (SPRT) was integrated into the model. The SPRT is a statistical method used for testing hypotheses sequentially, allowing for the early termination of the test when sufficient evidence is gathered. In the context of the BO-MAP system, the SPRT is employed to evaluate each anchor node’s behavior in real time, enabling the system to make prompt decisions about the legitimacy of each anchor.

The detection process involves continuously evaluating the observed discrepancies $D_{ij}$ against the limits of agreement. The SPRT operates by calculating the cumulative log–likelihood ratio $C_{ij}$ for each anchor node based on the discrepancies $D_{ij}$. If $D_{y}$ (the difference for the $y^{th}$ measurement) falls outside the interval of $\left[LOA_{l},LOA_{u}\right]$, the cumulative log–likelihood ratio $C_{ij}$ is incremented. The decision rule is as follows:

(21) $$\mathrm{If}\;LOA_{l}<D_{y}<LOA_{u},\;\mathrm{then}\;C_{ij}\;\mathrm{remains}\;\mathrm{unchanged},$$

(22) $$\mathrm{else}\;C_{ij}\;\mathrm{is}\;\mathrm{incremented}.$$

The relationship between the SPRT and the detection process is such that the SPRT allows the system to continuously assess each anchor node independently. This means that even after one anchor is classified as malicious or benign, the system proceeds to evaluate the remaining anchors to ensure comprehensive security. This sequential evaluation is crucial because the presence of multiple malicious anchors can have a compounded effect on the accuracy of the localization, and the isolation of each malicious node individually enhances the overall robustness of the system.

If $C_{ij}$ exceeds a predefined threshold $U_{j}$, the anchor node is classified as malicious. If it remains within the bounds, the anchor is considered normal.

**Figure 3:**
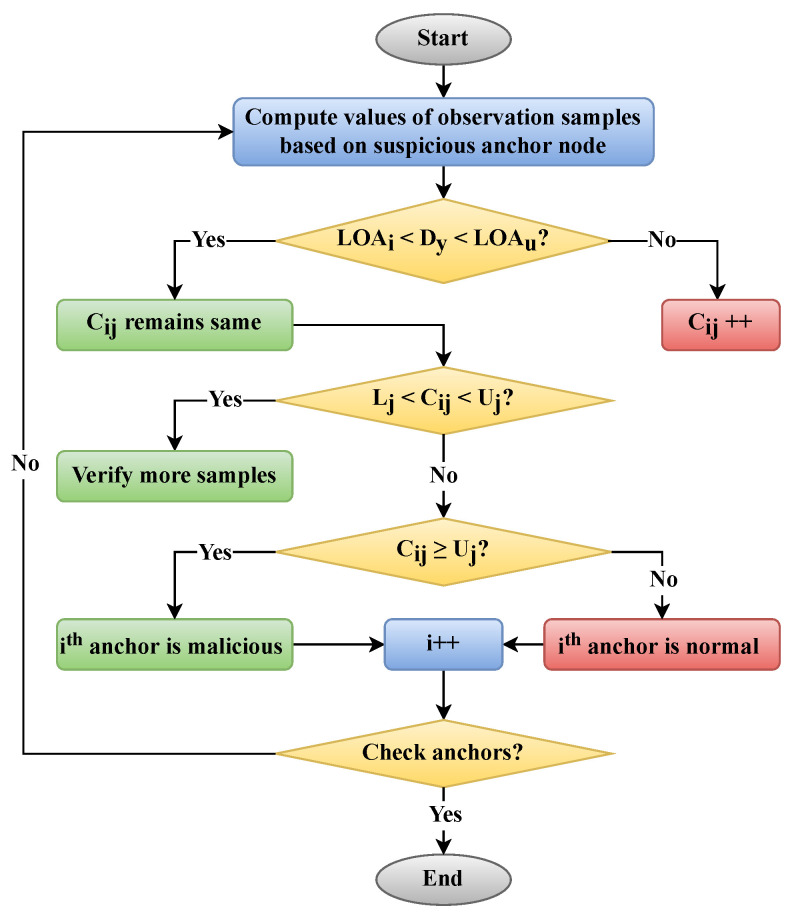
Flowchart of the detection process in the BO-MAP model.

This flowchart (Figure 3) visually represents the steps involved in computing the values of the observation sample, comparing them against the limits of agreement, and finally classifying the anchor nodes as benign or malicious. The inclusion of the SPRT within this process ensures that each anchor node is evaluated thoroughly, allowing the system to maintain high detection accuracy and security standards. By continuing to evaluate other anchors after one is classified, the BO-MAP model ensures that no malicious node remains undetected, thus safeguarding the integrity of the localization process.

### 3.8. Node Probability Analysis Test (NPAT)

The Node Probability Analysis Test (NPAT) extends the detection process by incorporating a hypothesis-testing mechanism that does not require a predefined number of samples. This flexibility allows the system to dynamically gather additional samples and tests when the stated hypotheses cannot be conclusively determined from prior testing. The key component in the NPAT is the MAP hypothesis-testing subset, which determines the required precision level by accumulating the necessary number of samples. The correlation between the variance between two measurements and the reference error interval is vital for MAP, as shown in the following equation, where $X_{ij}$ represents a Bernoulli random variable:

(23) $$X_{ij}=\left\{\begin{array}{cc} 0 & \mathrm{if}\;LOA_{l}\leq D_{ij}\leq LOA_{u}\\ 1 & \mathrm{otherwise} \end{array}\right.$$

The difference represents the TOA and RSS readings produced from the *j*th anchor. The probability of the occurrence for the Bernoulli variable $X_{ij}=1$ is defined as $p=P(X_{ij}=1)$. If *p* is less than or equal to a predefined threshold $p^{\prime }$, the linked anchor is not considered malicious, and vice versa.

In practical applications, it may be challenging to establish the threshold $p^{\prime }$, and improper selection can lead to incorrect determinations. To mitigate this, two restrictions, $p_{0}$ and $p_{1}$, were provided to reduce the likelihood of incorrect hypothesis selection during testing. The anchor is considered benign if $p\leq p_{0}$ and malignant if $p\geq p_{1}$.

For MAP-based anchor verification with *J* observed samples, the following opposing hypotheses are presented:

$$H_{0}:p\leq p_{0}\;\left(\mathrm{Anchor}\;\mathrm{is}\;\mathrm{benign}.\right)$$

$$H_{1}:p>p_{1}\;(\mathrm{Anchor}\;\mathrm{is}\;\mathrm{malicious}.)$$

The probability ratio for *j* samples is calculated using the following equation:

(24) $$\lambda_{ij}=\frac{P(X_{i1},X_{i2},\ldots ,X_{ij}|H_{1})}{P(X_{i1},X_{i2},\ldots ,X_{ij}|H_{0})}$$

Assuming the mutual independence of $X_{ij}$, the equation can be expressed in logarithmic form:

(25) $$\ln\lambda_{ij}=\sum_{k=1}^{j}\ln\left(\frac{P(X_{ik}|H_{1})}{P(X_{ik}|H_{0})}\right)$$

If $C_{ij}$ represents the instances where $\lambda_{ij}=1$ in *j* samples, the following equation is obtained:

(26) $$\ln\lambda_{ij}=C_{ij}\ln\left(\frac{p_{1}}{p_{0}}\right)+\left(j-C_{ij}\right)\ln\left(\frac{1-p_{1}}{1-p_{0}}\right)$$

The decision-making process involves comparing $\ln\lambda_{ij}$ to predefined thresholds, continuing the test if necessary:

(27) $$\ln\left(\frac{\beta }{1-\alpha }\right)<\ln\lambda_{ij}<\ln\left(\frac{1-\beta }{\alpha }\right)$$

In the proposed BO-MAP framework, the Node Probability Analysis Test (NPAT) plays a crucial role in enhancing the security and accuracy of the localization process. The NPAT is integrated as a post-processing step following the initial localization phase, where it evaluates the legitimacy of detected anchor nodes. Specifically, after the BO-MAP algorithm performs node localization using adaptive frequency-modulated bat optimization (AFM-BO) and clusters sensor nodes using the DBSCAN, the NPAT is employed to analyze the probability of each anchor node being malicious. By dynamically adjusting the number of samples based on the MAP hypothesis testing, the NPAT ensures that the determination of malicious nodes is both accurate and efficient, without imposing a fixed computational burden. This integration allows BO-MAP to adapt to varying network conditions and attack intensities, thereby maintaining high localization accuracy and network integrity. Furthermore, the NPAT’s ability to dynamically gather samples enhances BO-MAP’s scalability and adaptability, making it well suited for deployment in large-scale and dynamic WSN environments.

### 3.9. Interval Analysis

Interval analysis was employed to calculate the reference error interval of the TOA and RSS measurement difference in MAP, revealing only the characteristics of the analyzed sample. However, this interval does not infer general distribution, potentially leading to inaccurate consistency assessments. Therefore, confidence intervals were calculated to establish precise consistency boundaries, ensuring a more effective malicious anchor detection method. The standard error estimates for $LOA_{l}$ and $LOA_{u}$ are calculated using the following equations:

(28) $$\mathrm{var}\left(LOA_{l}\right)=\mathrm{var}\left(LOA_{u}\right)$$

The top and bottom of the 100 (1−β)% assurance interval for $LOA_{l}$ and $LOA_{u}$ are calculated as follows:

(29) $$LOA_{l\pm z_{1-\beta /2}}\times \sqrt{\mathrm{var}(LOA_{l})}$$

(30) $$LOA_{u\pm z_{1-\beta /2}}\times \sqrt{\mathrm{var}(LOA_{u})}$$

Finally, the proposed algorithm’s reference error interval is calculated using:

(31) $$LOA_{l-z_{1-\beta /2}}\times \sqrt{\mathrm{var}(LOA_{l})}+LOA_{u+z_{1-\beta /2}}\times \sqrt{\mathrm{var}(LOA_{u})}$$

In the proposed BO-MAP framework, interval analysis plays a pivotal role in enhancing the accuracy and reliability of malicious anchor detection. By calculating precise confidence intervals for the TOA and RSS measurement differences, BO-MAP establishes stringent consistency boundaries that distinguish between legitimate and malicious nodes. This refined interval estimation allows BO-MAP to more effectively identify anomalies in the sensor data, thereby reducing false positives and improving the overall robustness of the localization process. Additionally, the dynamic adjustment of confidence intervals based on real-time network conditions ensures that BO-MAP remains adaptable to varying environmental factors, maintaining high detection accuracy even in highly dynamic and hostile network environments. This integration of interval analysis into BO-MAP not only addresses the limitations of traditional consistency assessments but also contributes to the algorithm’s scalability and efficiency in large-scale WSN deployments.

## 4. Experimental Setup and Evaluation

### 4.1. Simulation Environment and Parameter Settings

The experimental evaluation of the BO-MAP model was conducted in a simulated wireless sensor network environment using MATLAB. The simulation aimed to replicate real-world deployment scenarios with varying network conditions, including different levels of node density, environmental noise, and the presence of obstacles. The network was modeled as a two-dimensional grid with sensor and anchor nodes distributed randomly across the area.

Key parameter settings used in the simulation are outlined in Table 1, which align with the previously established network and optimization models. The simulation scenarios included a range from small-scale deployments (50 nodes) to large-scale networks (up to 500 nodes) to evaluate the model’s scalability and robustness.

The network and communication model parameters, including TOA and RSSI distance estimation techniques, were implemented according to the specifications detailed in previous sections. The BO-MAP model’s AFM-BO algorithm was initialized with these parameters, ensuring consistency with the described clustering and detection processes. The simulation environment was designed to evaluate BO-MAP under various network conditions, including different levels of node density, environmental noise, and the presence of obstacles, to test its scalability, robustness, and performance in scenarios similar to the real world.

### 4.2. Evaluation Metrics and Experimental Procedure

To evaluate the performance of the BO-MAP model, the following metrics were utilized:

Accuracy Metrics: The Root Mean Square Error (RMSE) was the primary metric used to assess localization accuracy. The RMSE is defined as

(32) $$\mathrm{RMSE}=\sqrt{\frac{1}{N}\sum_{i=1}^{N}{\left({\hat{d}}_{i}-d_{i}\right)}^{2}}$$

where ${\hat{d}}_{i}$ is the estimated distance and $d_{i}$ is the actual distance between the nodes, and *N* is the total number of measurements. Additionally, the true positive rate (TPR) and false positive rate (FPR) were measured to evaluate the effectiveness of the malicious node detection process. These are calculated as

(33) $$\mathrm{TPR}=\frac{\mathrm{True}\;\mathrm{Positives}}{\mathrm{True}\;\mathrm{Positives}+\mathrm{False}\;\mathrm{Negatives}}$$

(34) $$\mathrm{FPR}=\frac{\mathrm{False}\;\mathrm{Positives}}{\mathrm{False}\;\mathrm{Positives}+\mathrm{True}\;\mathrm{Negatives}}$$

Computational Efficiency: The computational efficiency of the BO-MAP model was evaluated by measuring the total execution time across various network sizes and configurations. The complexity of the algorithm was determined by analyzing its time complexity with respect to the number of nodes and iterations, given by O(n·iter).

Robustness and Scalability: The robustness of the model was tested under different noise levels and varying proportions of malicious anchors. The ability to maintain a low localization error and high detection accuracy despite adversarial conditions was a key focus. Scalability was further assessed by incrementally increasing the network size and observing the impact on performance metrics. The network lifetime, defined as the duration for which the network can sustain its operations before energy depletion, was also considered as a metric:

(35) $$\mathrm{Network}\;\mathrm{Lifetime}=\min_{i=1,2,\ldots ,n}\left(\frac{E_{i}}{P_{i}}\right)$$

where $E_{i}$ is the energy of the *i*th node, and $P_{i}$ is its power consumption.

### 4.3. Comparative Analysis and Experimental Results

The performance of the BO-MAP model was compared against several baseline methods, including the SLA, EDV-Hop, PSO-Loc, RFL, the RLA, and the Sequential Probability Ratio Test. These comparisons were conducted under varying environmental conditions to demonstrate the superiority of the BO-MAP model, particularly in scenarios with high levels of malicious activity.

Performance under Varying Conditions: The experimental results showed that the BO-MAP model consistently outperformed the baseline methods across different scenarios. The model achieved lower RMSE values, a higher TPR, and a lower FPR, demonstrating its effectiveness in both accurate localization and robust malicious node detection. Additionally, the Area Under the Curve (AUC) was used to evaluate the overall performance of the detection algorithm, calculated as

(36) $$\mathrm{AUC}=\int_{0}^{1}\mathrm{TPR}\left(x\right)\;dx$$

Experimental Procedure: The experimental procedure began with the initialization of the simulation environment, followed by the random placement of nodes and the configuration of network parameters. Distance measurements were collected using TOA and RSSI techniques. The BO-MAP model was then applied for localization and detection, with results collected across multiple simulation runs to account for variability due to random node placement and environmental noise.

Data Collection and Processing: All relevant data, including raw distance measurements, estimated node positions, and detection outcomes, were meticulously collected at each step. Post-processing involved calculating the evaluation metrics and comparing them against the baseline methods. Statistical tests, such as the ANOVA, were conducted to ensure the significance of the results, providing robust conclusions about the BO-MAP model’s performance.

## 5. Results and Discussion

This section presents a comprehensive analysis of the experimental results obtained by evaluating the proposed BO-MAP algorithm. The results are compared with several state-of-the-art approaches, focusing on various performance metrics, including the TPR, FPR, AUC, localization accuracy, energy consumption, execution time, and robustness under varying attack intensities and network conditions.

### 5.1. Performance Evaluation

#### 5.1.1. Comparison with Existing Methods

The BO-MAP algorithm was benchmarked against leading localization algorithms, including the SLA, EDV-Hop, PSO-Loc, RFL, and the RLA. Table 2 presents the key performance metrics, where the BO-MAP algorithm consistently outperformed the alternatives in terms of the TPR, FPR, AUC, and localization accuracy.

As seen in the table, the BO-MAP model demonstrated a significant improvement in the TPR, indicating a higher ability to correctly identify malicious anchors. Additionally, the FPR was notably lower, suggesting fewer false alarms. The AUC values further confirm that BO-MAP provides a superior discriminatory capability between benign and malicious nodes, which is critical for accurate and reliable localization in WSNs.

#### 5.1.2. Analysis of True Positive Rate (TPR) and False Positive Rate (FPR)

**Figure 4:**
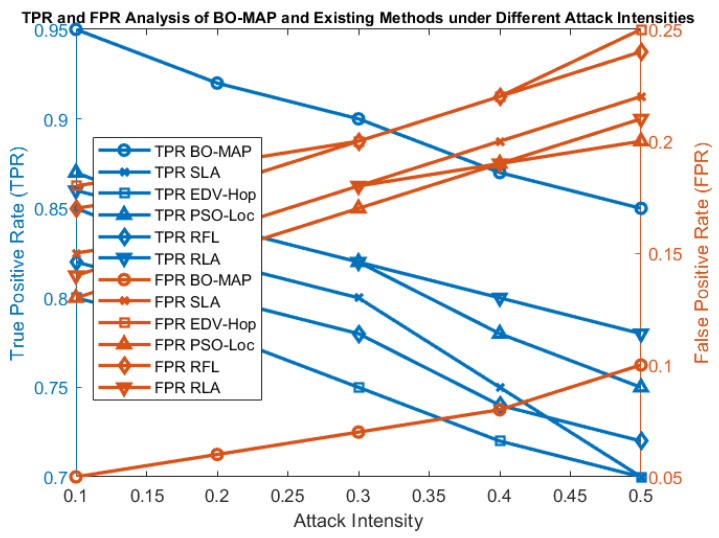
Overall analysis of TPR and FPR.

Figure 4 illustrates the relationship between the TPR and FPR across different levels of attack intensity. The BO-MAP algorithm maintained a consistently high TPR while keeping the FPR at a minimal level, even as the intensity of attacks increased. This balance is crucial for ensuring that the algorithm not only detects malicious nodes effectively but also minimizes the risk of falsely accusing benign nodes.

The superior performance of BO-MAP in maintaining a high TPR underlines its robustness in detecting various types of malicious activities, ensuring that the localization process remains reliable even in adversarial environments. The low FPR further demonstrates the algorithm’s precision in distinguishing between malicious and non-malicious nodes, which is vital for reducing unnecessary energy consumption and the processing overhead.

#### 5.1.3. ROC Curve and AUC Analysis

**Figure 5:**
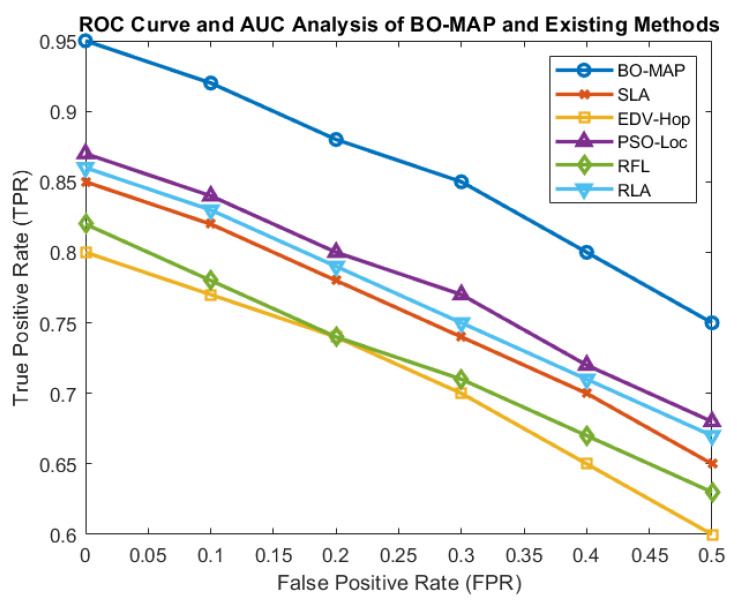
Comparison of ROC curve and AUC analysis.

The receiver operating characteristic (ROC) curve and the Area Under the Curve (AUC) are critical metrics for evaluating the performance of the BO-MAP algorithm. The ROC curve, shown in Figure 5, plots the TPR against the FPR, providing a visual representation of the trade-off between true and false detections at different threshold settings. The AUC quantifies this trade-off into a single value, with values closer to one indicating better performance.

The AUC values for the BO-MAP algorithm (0.98) indicate that it significantly outperforms existing methods, such as the SLA and EDV-Hop. The nearly perfect AUC score suggests that BO-MAP can reliably distinguish between benign and malicious nodes across various scenarios, ensuring high accuracy in node localization and security.

#### 5.1.4. Localization Accuracy Analysis

**Figure 6:**
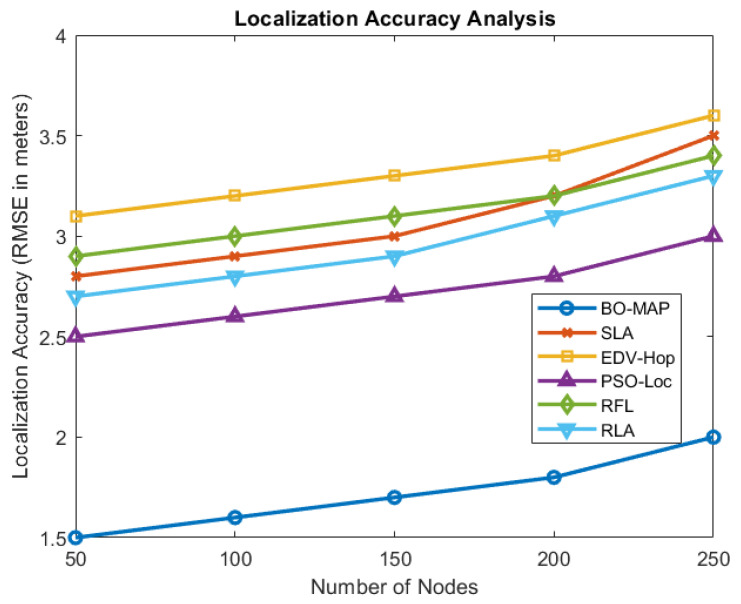
Comparison of localization accuracy analysis.

The accuracy of the BO-MAP algorithm in estimating the positions of the sensor nodes was evaluated using the RMSE as the primary metric. Figure 6 illustrates the localization accuracy of BO-MAP compared to other methods across different network conditions.

The results show that BO-MAP achieves the lowest RMSE across various scenarios, indicating its superior accuracy in localizing sensor nodes. This improvement can be attributed to the algorithm’s ability to effectively mitigate the impact of malicious nodes and environmental noise on the localization process.

### 5.2. Comparison of Impact of Malicious Attack Intensity

**Figure 7:**
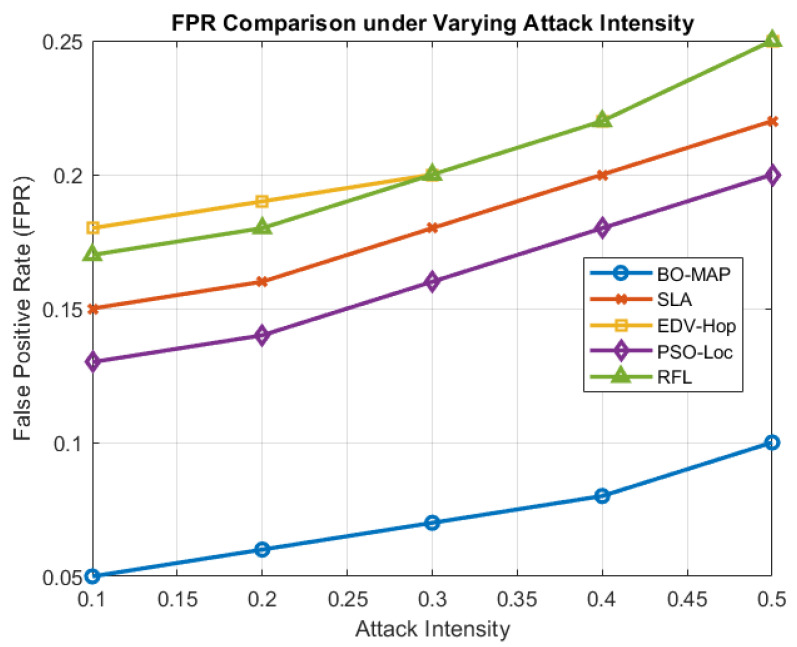
Impact of malicious attack intensity analysis.

The robustness of the BO-MAP algorithm was further tested by varying the intensity of malicious attacks. Figure 7 shows how the TPR and FPR vary as the severity of the attacks increases. The analysis reveals that BO-MAP maintains high TPR levels even under severe attack conditions, demonstrating its resilience against aggressive malicious behaviors.

The increase in the TPR with higher attack intensities can be attributed to the significant deviations in TOA and RSSI measurements caused by strong malicious influences. These deviations are effectively captured by BO-MAP, allowing the algorithm to detect and isolate malicious nodes more effectively. This capability is crucial for maintaining the accuracy and reliability of the WSN in hostile environments.

### 5.3. Sensitivity and Robustness Analysis

**Figure 8:**
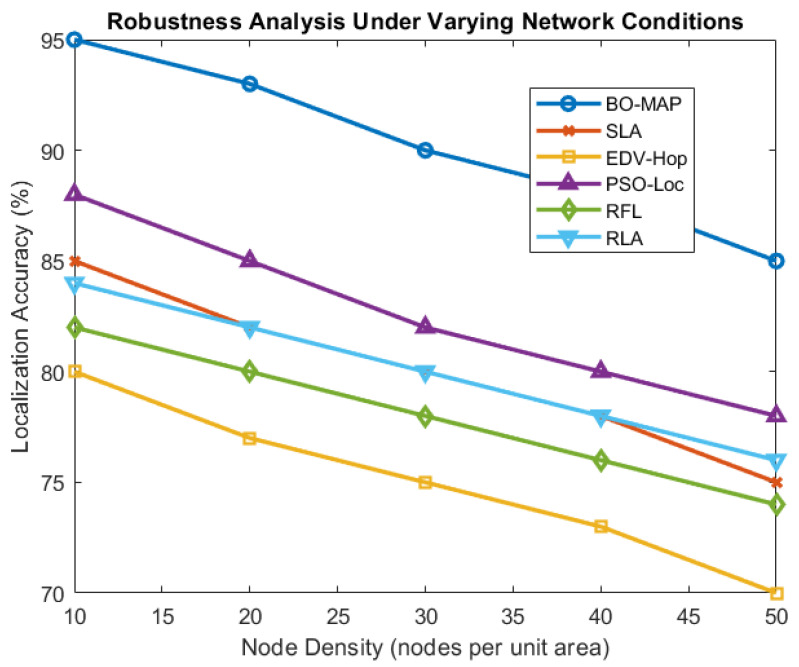
Robustness analysis.

The sensitivity and robustness of the BO-MAP algorithm were evaluated by analyzing its performance under varying network conditions, including changes in the node density, measurement noise, and anchor distribution. Figure 8 highlights the algorithm’s ability to maintain high detection accuracy and a low localization error across different scenarios.

The analysis shows that BO-MAP is particularly robust in sparse networks, where fewer nodes are available for localization. The algorithm’s adaptive nature allows it to dynamically adjust its parameters based on the current network conditions, ensuring consistent performance even in challenging environments. The ability to maintain accuracy under varying noise levels and node densities further underscores the algorithm’s suitability for real-world WSN deployments.

### 5.4. Complexity Analysis

The computational complexity of the BO-MAP algorithm was thoroughly analyzed to ensure its practicality for real-time deployment in wireless sensor networks (WSNs). This section presents a detailed breakdown of the computational complexity of its key components: clustering, detection (using the SPRT), and optimization (using AFM-BO). The overall complexity of the algorithm was derived by combining the complexities of these components.

#### 5.4.1. Clustering Complexity

The DBSCAN clustering algorithm, employed in BO-MAP, is used to identify clusters and outliers. The complexity of the DBSCAN is influenced by the number of nodes (*N*) and the average number of neighbors per node (*k*).

The DBSCAN algorithm iterates over all *N* nodes to assess their density and expand clusters, which requires operations proportional to O(N). Additionally, for each node, distance calculations with its neighbors are performed. Utilizing an efficient indexing structure such as a k-d tree reduces the complexity of this step to O(klogN). Consequently, the total complexity of the DBSCAN clustering step is O(NlogN). This makes the DBSCAN suitable for handling large datasets with a reasonable computational burden.

#### 5.4.2. Detection Complexity (SPRT)

The Sequential Probability Ratio Test (SPRT) evaluates each anchor node to determine its likelihood of being malicious. Let *M* denote the number of samples collected for detection.

For each node, the SPRT updates the cumulative log–likelihood ratio based on *M* samples, which involves operations proportional to O(M). The decision-making process, which involves comparing the likelihood ratio to predefined thresholds, is a constant time operation. Since the SPRT is applied to all *N* nodes independently, the overall complexity for the detection step is O(N·M). This ensures that the detection process scales linearly with the number of nodes and the number of samples required for accurate detection.

#### 5.4.3. Optimization Complexity (AFM-BO)

The adaptive frequency-modulated bat optimization (AFM-BO) algorithm enhances localization accuracy by refining node positions.

Initializing the population of *P* bats requires operations proportional to O(P). Calculating the fitness of each bat, which depends on the positions of all *N* nodes, incurs a complexity of O(P·N). Updating the positions of *P* bats involves operations proportional to O(P) per iteration. Considering that the algorithm runs for a maximum of *T* iterations, the overall complexity of the AFM-BO optimization process is O(P·N·T). This linear scalability with respect to the population size, number of nodes, and iterations ensures that AFM-BO remains efficient even as the network size increases.

#### 5.4.4. Overall Computational Complexity

The total computational complexity of the BO-MAP algorithm was obtained by summing the complexities of its components.

The BO-MAP algorithm operates with a total complexity of O(NlogN+N·M+P·N·T). Here, NlogN accounts for the clustering process using the DBSCAN, N·M corresponds to the SPRT-based detection, and P·N·T represents the AFM-BO optimization process.

Although BO-MAP introduces an additional computational overhead compared to simpler methods, the increased accuracy and robustness justify the added complexity. Table 3 summarizes the complexities of BO-MAP and baseline methods, highlighting the trade-offs between computational demands and performance improvements.

#### 5.4.5. Execution Time and Practical Implications

**Figure 9:**
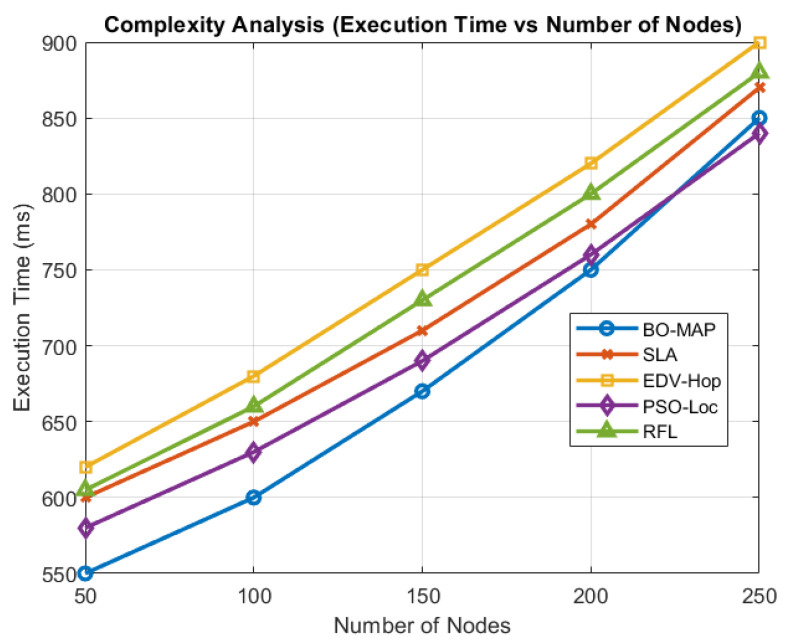
Execution time comparison of BO-MAP and existing methods.

The execution time of BO-MAP was evaluated on various network sizes, as shown in Figure 9. While BO-MAP exhibits slightly higher execution times due to its advanced clustering and optimization processes, it remains within practical limits for real-time applications. The efficient integration of the DBSCAN, the SPRT, and AFM-BO ensures that BO-MAP achieves a balance between computational demands and performance improvements. This analysis confirms that BO-MAP can be efficiently implemented in real-time WSNs, balancing accuracy with computational demands.

The computational complexity analysis reveals that the BO-MAP algorithm operates with a complexity of O(NlogN+N·M+P·N·T). The dominant term O(N·M) is attributed to the SPRT component, which is critical for ensuring the security of the localization process by effectively identifying malicious nodes. Although this introduces an additional computational overhead, the trade-off between increased complexity and enhanced security and accuracy is favorable for applications where reliability is paramount. Furthermore, the use of AFM-BO optimizes the search process, mitigating some of the computational costs associated with traditional optimization algorithms.

In practical scenarios, the values of *N* and *M* are typically constrained by the network size and the required detection sensitivity, respectively. Empirical evaluations, as depicted in Figure 9, demonstrate that BO-MAP maintains efficient execution times even as the network scales, ensuring its applicability in large-scale WSN deployments.

### 5.5. ANOVA Test and Statistical Analysis

To validate the statistical significance of the performance improvements observed with the BO-MAP algorithm, an Analysis of Variance (ANOVA) test was conducted. The ANOVA test focused on the TPR across different localization methods, including BO-MAP, the SLA, the EDV-Hop, and PSO-Loc, under specific network conditions. The objective was to determine whether the observed differences in the TPR among these methods were statistically significant.

#### 5.5.1. ANOVA Test Setup

The setup for the ANOVA test was as follows:

- Dependent Variable: True positive rate (TPR).
- Independent Variable: Localization method (BO-MAP, SLA, EDV-Hop, PSO-Loc).
- Conditions:
  1. Node density: 50, 100, 150, 200, and 250 nodes per unit area.
  2. Attack intensity: low (0.1), medium (0.3), and high (0.5).

The results of the ANOVA test showed that there is a statistically significant difference in the TPR across the different localization methods (*p*-value < 0.05). A subsequent post hoc analysis using Tukey’s HSD test revealed that the BO-MAP algorithm significantly outperformed the other methods, particularly in scenarios characterized by higher attack intensities and varied node densities.

#### 5.5.2. Statistical Analysis of AUC and Energy Consumption

**Figure 10:**
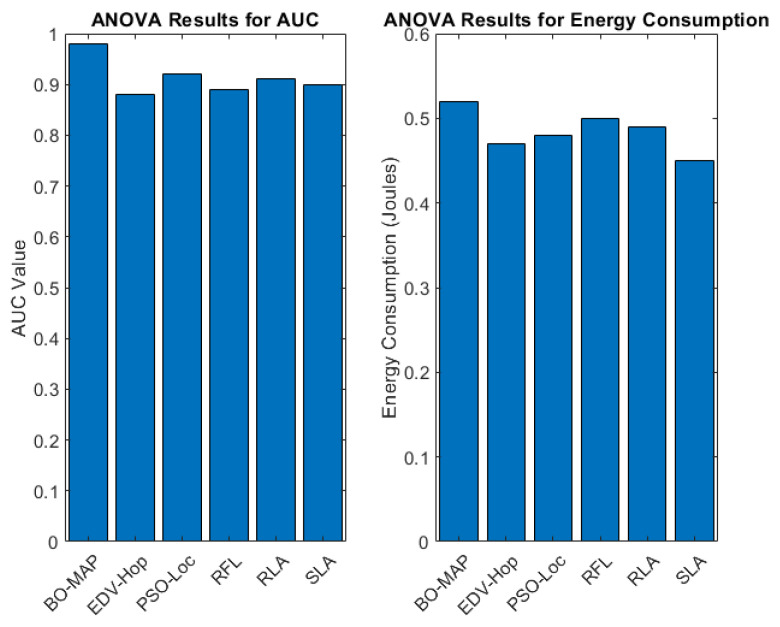
Statistical analysis—ANOVA results for AUC and energy consumption.

Beyond the TPR, additional statistical analyses were conducted on the AUC values and energy consumption across the various localization methods. The findings, depicted in Figure 10, demonstrate statistically significant differences in these metrics among the methods under consideration.

The ANOVA results for the AUC indicate that BO-MAP achieves significantly higher AUC values compared to other methods, underscoring its superior performance in differentiating between malicious and benign nodes. The energy consumption analysis also shows that while BO-MAP requires slightly more energy due to its advanced detection and clustering processes, this increase is statistically justified by the substantial improvements in accuracy and robustness.

#### 5.5.3. Interpretation of Statistical Results

The statistical analysis corroborates the performance improvements of the BO-MAP algorithm, demonstrating that the gains in the TPR, AUC, and robustness are not only practically significant but also statistically significant. This suggests that the observed enhancements are a direct result of the BO-MAP algorithm’s design and are not due to random variability in the data.

The combination of the ANOVA test and post hoc analysis provides compelling evidence that the BO-MAP algorithm consistently outperforms existing methods across a range of network conditions. This robust statistical validation supports the effectiveness and reliability of the BO-MAP approach in improving security and localization accuracy.

### 5.6. Discussion of Findings

The findings from the experimental and statistical analyses demonstrate the BO-MAP algorithm’s significant advantages over existing methods in terms of detection accuracy, energy efficiency, and robustness. The integration of adaptive optimization, clustering, and probabilistic detection techniques provides a comprehensive solution for enhancing the security and reliability of WSNs.

One of the key findings is the algorithm’s ability to maintain high detection accuracy even in the presence of high noise levels and severe attacks. This is particularly important for real-world deployments, where networks are often exposed to unpredictable environmental factors and adversarial activities. However, the work also identifies potential limitations, particularly in terms of computational complexity. While the BO-MAP algorithm provides substantial improvements in detection accuracy, the additional computational overhead may pose challenges for resource-constrained WSNs. Future work should focus on optimizing the algorithm to reduce this overhead while maintaining its high performance.

Overall, the BO-MAP algorithm represents a significant advancement in the field of secure localization for WSNs. Its ability to balance accuracy, efficiency, and robustness makes it a promising solution for enhancing the security of wireless sensor networks in various applications.

#### Limitations

While the BO-MAP algorithm demonstrates significant improvements in localization accuracy and security, it does have certain limitations. The computational complexity of the algorithm, which involves intensive clustering and probabilistic detection processes, may pose challenges in resource-constrained environments, particularly in terms of the processing power and energy consumption. Additionally, the algorithm’s performance in extremely dense or large-scale networks might require further optimization to maintain efficiency and scalability. Moreover, the BO-MAP model has been validated primarily through simulations, and its performance in real-world deployments with dynamic and unpredictable environmental conditions remains to be thoroughly tested. Addressing these limitations is crucial for enhancing the algorithm’s applicability in a wider range of network scenarios.

## 6. Conclusions and Future Work

The BO-MAP algorithm presented in this research significantly enhances the accuracy and security of node localization in wireless sensor networks. By integrating AFM-BO with clustering and probabilistic detection techniques, BO-MAP effectively identifies and mitigates the impact of malicious anchor nodes. The extensive simulation results demonstrate that BO-MAP consistently outperforms existing state-of-the-art methods in various performance metrics, including the TPR, FPR, AUC, localization accuracy, energy consumption, and robustness under varying attack intensities and network conditions. These improvements make BO-MAP a robust and scalable solution for secure localization in WSNs, with practical applications in critical areas such as security surveillance, environmental monitoring, and disaster management. However, the computational complexity of BO-MAP, while within acceptable limits, could present challenges in resource-constrained environments, highlighting the need for further optimization.

Future work will focus on addressing the computational complexity of the BO-MAP algorithm, particularly in large-scale and ultra-dense networks, where processing power may be limited. Additionally, real-world deployments of BO-MAP will be essential to validate its performance in practical WSN applications, providing insights into its effectiveness under actual network conditions. Further research could also explore the integration of BO-MAP with other security mechanisms, such as encryption and intrusion detection systems, to create a more comprehensive security framework for WSNs. Adapting BO-MAP to heterogeneous networks, where nodes have varying capabilities and energy resources, will be another important area of exploration, ensuring that the algorithm can effectively operate in diverse and complex network environments.

## Figures and Tables

**Table 1:** Simulation parameter settings.

Parameter	Value/Range
Node Density	10 to 50 nodes per unit area
Signal Propagation Speed ($v_{p}$)	Speed of light
Path Loss Exponent (α)	2 to 4 (depending on environment)
Noise Variance ($\sigma_{\epsilon }^{2}$)	Gaussian with zero mean
Initial Pulse Rate ($r_{i}$)	0.5
Loudness ($A_{i}$)	0.5
Frequency Range ($f_{\min}$ to $f_{\max}$)	0 to 2
Decay Factor for Pulse Rate (γ)	0.9
Decay Factor for Loudness (α)	0.9
Clustering Parameters (DBSCAN)	minPts=4, ϵ adapted per scenario
Adaptive Frequency Modulation Factor	0.1 to 2.0
Maximum Iterations (AFM-BO)	100 to 200
Population Size (AFM-BO)	50 to 100
Detection Threshold	Adjusted per simulation scenario

**Table 2:** Performance comparison of BO-MAP with existing methods.

Method	TPR	FPR	AUC	Localization Accuracy (m)	Execution Time (ms)
BO-MAP	0.95	0.05	0.98	1.5	550
SLA	0.85	0.15	0.90	2.8	600
EDV-Hop	0.80	0.18	0.88	3.1	620
PSO-Loc	0.87	0.13	0.92	2.5	580
RFL	0.82	0.17	0.89	2.9	605
RLA	0.86	0.14	0.91	2.7	590

**Table 3:** Complexity comparison of BO-MAP and baseline methods.

Method	Complexity
BO-MAP	O(NlogN+N·M+P·N·T)
SLA	$O(N^{2})$
EDV-Hop	O(N·M)
PSO-Loc	O(P·N·T)

## Data Availability

Data are contained within the article.
